# Home Hemodialysis (HHD) Treatment as an Effective yet Underutilized Treatment Modality in the United States

**DOI:** 10.3390/healthcare5040090

**Published:** 2017-11-28

**Authors:** Jihane J. Hajj, Krzysztof Laudanski

**Affiliations:** 1 School of Nursing, Widener University, One University Pl, Chester, PA 19013, USA; jjhajj@widener.edu; 2 Department of Anesthesiology and Critical Care Medicine, University of Pennsylvania, 3650 Hamilton Walk, Philadelphia, PA 19146, USA

**Keywords:** home hemodialysis, quality of life, health economy, behavioral medicine, outcomes

## Abstract

End-stage renal disease (ESRD) is a major health burden and its incidence has been increasing yearly reaching 120,000 cases in 2014. Home hemodialysis (HHD) is a treatment modality option that has been shown to contribute to numerous clinical benefits but is largely underutilized due to many contributing factors. The purpose of this review paper is to analyze the advantages and disadvantages of HHD and the reasons for its low utilization with a special focus on its socioeconomic impact as compared to facility hemodialysis. Key factors contributing to HHD underutilization are related to the reimbursement system of the facility and nephrologists as well as the underutilization of the pre-dialysis educational benefit. Based on this comprehensive review of the literature, we propose several suggestions which may contribute to the expansion of HHD treatment modality.

## 1. Introduction

Chronic Kidney Disease (CKD) remains a public health problem that affects around 14% of the American population. The increase in life expectancy coupled with the rise in the incidence of chronic illnesses contributes to the worsening of the CKD state, leading to its advanced stage—namely End Stage Renal Disease (ESRD). ESRD cases have been increasing at an average of 2.5% annually since 1996 and in 2014, the number of incident cases of ESRD in the US was approximately 120,000 making a total of around of 470,000 dialysis patients in the US [1,2].

ESRD patients face a lifelong burden and the nature of this burden is very much dependent on the treatment modality. Home hemodialysis (HHD) may lessen the patient’s disease burden and improve quality of life while preserving clinical measures of success but it remains underutilized. The purpose of this review is to analyze the advantages and disadvantages of HHD and the reasons for its low utilization. We specifically focused on the current state of HHD utilization, factors influencing its underutilization, its socioeconomic impact as compared to facility hemodialysis and the role it plays in improving patient satisfaction, survival and quality of life.

Medicare set up a goal of 25% utilization of HHD for ESRD patients but current data demonstrates that this aim is far from reaching the goal. HHD remains underutilized accounting for 3.4% of home dialysis patients despite 120% increase in utilization between 2007 and 2016 [2]. This underutilization begs the investigation of its underlying reasons. Additionally, we would like to understand the socio-economic benefits of HHD.

## 2. Materials and Methods

This was a comprehensive review of the literature of HHD utilization in the US. The databases utilized were Pubmed, Medline, Google Scholar. The web pages of the Center for Medicare and Medicaid Services, National Kidney Foundation and US Renal Data System report were also utilized for this review. A wide and comprehensive list of subject headings were used, namely, “home hemodialysis,” “conventional hemodialysis,” “cost effectiveness,” “pre-dialysis education,” “Medicare reimbursement,” “quality of life,” “clinical benefits,” and “survival.” We restricted the search to manuscripts written in English and to original studies that were peer-reviewed and were conducted in the United States except for those studies that examined the role of pre-dialysis education on the selection of treatment modality as this topic could be universally applied. Studies completed outside of the US were excluded as they may not deem relevant to the purpose of this review.

Using the keywords mentioned earlier, a total of 833 articles were retrieved. A total of 24 papers met the inclusion and exclusion criteria of this review. The inclusion criteria for the papers selected were as follow: (1) Papers were written in English language; (2) Papers were original studies whether meta-analysis or original research studies; (3) Research studies completed on US patients or in US-based dialysis centers except for research that examined the role of pre-dialysis education on the selection of treatment modality as this topic could be universally applied. Studies completed outside the US were therefore excluded as these may not deem relevant to the purpose of the study.

The result of this literature review led to the emergence of different themes that explain the nature of HHD utilization and factors affecting its underutilization. These included the nature of Medicare reimbursement to providers and facilities and the role of pre-dialysis education. Given that HHD has been shown to be cost-effective and is associated with numerous clinical benefits, we found it to be an important addition to the discussion that supports the purpose of this review.

## 3. Results

### 3.1. Economics of Renal Replacement Therapy (RRT)

ESRD is a disease condition that is covered by Medicare regardless of the individual’s age or disability status. ESRD population accounts for less than 1% of the Medicare beneficiaries but utilizes around 7% of Medicare fee-for-service spending or around $33 billion. This constitutes a disproportionate share of the overall Medicare spending which is primarily reflecting the increase in the number of patients [3]. Following the implementation of the bundled payment system (PPS), the fee for service of ESRD spending continued to grow modestly by around 3.3% in the year 2013–2014. Several modes of dialysis can be offered in variety settings and applying certain policies may affect the changes long-term. However, early research completed couple years after the PPS implementation demonstrated a modest increase in the use of Peritoneal dialysis (PD) modality but there was no effect on HHD [4]. This is in striking contrast to Medicare stakeholders estimating that 50% of ESRD patients could be eligible for home dialysis. However, they set a more reasonable target of 25% which has not been attained [5]. Many impediments that may currently preclude the increase in the use of home hemodialysis treatment; and these are: (1) the nature of Medicare reimbursement to facilities; (2) the nature of Medicare reimbursement to physicians; and (3) the utilization of the Kidney Disease Education (KDE) benefits.

### 3.2. Nature of Medicare Reimbursement to Facilities

Medicare has the same bundled payment that applies to all three treatment modalities (in-center hemodialysis, HHD and PD). In other words, Medicare pays around $230 per treatment for up to three treatments per week regardless of the modality of renal replacement therapy (RRT). However, reimbursement to facilities defer for HHD patients because Medicare pays an adjusted rate which includes the cost of training or around $50 per treatment for up to 25 treatments which is equivalent to 37.5 h and $1881 [6]. Also, Medicare reimburses HHD treatments (for patients under age of 65) from the first day unlike the in-center HD which starts in the fourth month. Despite these financial incentives, HHD could be expensive in the short term and these incentives are not sufficient for facilities to encourage the increase utilization of home HD. In fact, the addition of a patient for in-center HD will not necessarily incur the addition of a dialysis machine which typically could be used on around 7 patients but a HHD therapy utilizes a machine exclusively assigned to one patients. Consequently, HHD is more expensive and less appealing to facilities to support given that patients utilizing this home modality tend to self-dialyze more frequently than three times per week, their machine is not shareable and reimbursed and capped at three sessions weekly. Also, in the short term, facilities benefit from in-center HD as this tends to increase the facilities’ Medicare margins estimated at 4% compared to home HD which has no effect on Medicare margins [5].

### 3.3. The Nature of Medicare Physician Reimbursement

Medicare reimburses nephrologists who provide care for HHD patients slightly differently than in-center patients with target to boost home HD utilization. As a way to achieve their goal in increasing the amount of HHD treatments, nephrologists who supervise home dialysis training are offered a one-time payment of $500. Medicare pays a monthly rate to nephrologists for caring for HD patients between $184 for one visit up to $282 for four or more visits. Nephrologists who care for HHD patients are required to complete at least one face to face visit which includes a complete assessment of the patient as well as assessment of the vascular site. This is reimbursed at $237 monthly compared to $266 for in-center patients. In reality, this plan is a disincentive to nephrologists. While nephrologists are required to have at least a one face to face visit, they are still required to continue monitoring the care of home HD patients throughout the week which is time consuming and not reimbursable at the same rate of in-center HD patients. Also, nephrologists would be able to complete and get reimbursed for multiple in-center patient visits on a single day as compared to one individual home patient visit in a private setting [5].

### 3.4. Kidney Disease Education (KDE) Benefit

Increasing the use of HHD modalities aims at fostering patient independence and autonomy which potentially has a positive impact on quality of life as well as other clinical benefits.

As a way to encourage the increased utilization of HHD, Medicare stressed the importance of providing pre-dialysis education to help patients with informed treatment decisions. Medicare Improvements for Patients and Providers Act of 2008 reimbursed nephrologists for providing pre-dialysis education for stage IV CKD patients who are anticipated to be on RRT. The education provided aims mainly at discussing the different treatment modalities available with the anticipation that a greater number of patients will choose a home modality when provided with such education [5]. There is a great amount of literature that supports this latter provision [7,8,9,10,11]. For example, Little and Colleagues (2001) examined the factors that influence patients’ choice of dialysis modalities. They found that counseling before dialysis favors choosing home dialysis rather than conventional HD. Such finding led to the implementation of a free choice policy at the renal department at Birmingham heartlands hospital which led to a steady growth in the amount of home dialysis by around 15% per year. Another multivariate analysis study examined determinants of modality selection. Data examined were derived from the Dialysis Morbidity and Mortality Wave 2 study which consisted of around 4000 patients. This study demonstrated that Pre-ESRD care was a major contributor to the selection of a home dialysis option which included patient counseling, education and autonomy [10]. Despite all these data, it was estimated that less than 2% of eligible ESRD patients received the educational benefits in 2010–2011 [12]. One contributing factor that may explain the underutilization of the educational benefits is the fact that nephrologists do not receive extensive training on home dialysis. Studies have shown that around 15% of nephrologists felt comfortable caring for HHD patients and those who were comfortable caring for home dialysis patients tend to prescribe it to a greater degree [13,14]. Also, the reimbursement amount may not be sufficient.

### 3.5. Cost-Effectiveness of Home Hemodialysis

Several studies have demonstrated the cost-effectiveness of home dialysis therapy. In the first published economic evaluation of dialysis modality conducted by Klarman and Colleagues in 1968 [15], a $66,000 difference in the per capita cost of HHD as compared to conventional HD ($38,000 versus $104,000 respectively) was seen. The cost-effectiveness ratio (the difference in cost of HHD and conventional HD divided by the difference in their effect) was $4200 for HHD as compared to $11,000 for conventional HD. In the follow-up studies that compared the cost-effectiveness of the various dialysis modalities, in-center HD was the most cost inefficient [16]. Lee and Colleagues (2002) [17] reported an annual cost of care difference of $21,000 ($51,252 for in-center HD versus $29,961 for HHD; *p* < 0.001) in a very robust study. Finally, the general conclusions regarding cost-effectiveness of HHD was reconfirmed in a systematic review of 6 newer studies which continued to demonstrate the cost-effectiveness of HHD over conventional HD [18].

### 3.6. Effect of Home Hemodialysis on Quality of Life and Clinical Outcomes

In theory, HHD should offer more favorable clinical, survival and quality of life outcomes. In a randomized control trial that involved around 240 patients (randomized into conventional facility and frequent home hemodialysis), patients in the frequent home hemodialysis demonstrated an improved control of hypertension, a significant increase in the physical health composite score (3 vs. 0.2; *p* = 0.004) and a significant decrease in the left ventricular mass (16 vs. 2 gms; *p* < 0.001) [19]. Similarly, a systematic review of 14 studies conducted by Walsh and Colleagues (2005) [20] demonstrated that frequent nocturnal hemodialysis was associated with a decreased use of antihypertensive medications, decrease in left ventricular mass at 12 months, increased hemoglobin levels and improved health-related quality of life in its subscale scores. Mowatt and Colleagues (2004) [21] conducted a systematic review that examined 27 studies which included 1 RCT 4 systematic reviews and concluded the following. Most recently, a systemic review was conducted to examine the evidence of nocturnal hemodialysis as compared to conventional hemodialysis. This study demonstrated that patients who underwent nocturnal hemodialysis had an improved blood pressure and hemoglobin levels compared to conventional hemodialysis [22]. When compared to hospital hemodialysis, home hemodialysis had on average 43% lower mortality rate than hospital hemodialysis and the risk of death is lower during the first 18 months of receiving dialysis among the home hemodialysis group. The etiology of this finding is believed to be related to the health status of candidates and other pertinent demographic data. For example, HHD candidates tend to be younger, independent and have fewer comorbidities. This is a typical pattern seen in patients enrolled in therapies with more activity on their side. Level of anemia is important variable defining mortality and quality of life among ESRD patients on RRT. Patients who received home hemodialysis had higher hemoglobin, hematocrit levels and required a 41% less of erythropoietin dosage compared to short 4-h hospital HD [21]. Considering the significant expenses related to EPO decreased demand is an added benefit for the cost containment measures offered by HHD. Studies demonstrated that HHD patients are more likely to be employed compared to in-center dialysis patients. This surely contributes to a lower burden on the society and may indirectly contribute to improving the quality of life of the affected individuals. However, these factors are frequently not taken into account by the insurance company and reflected in their payment structure. Finally, patients who undergo HHD experienced less of adverse events. In fact, they experience few incidences of hypotension, arrhythmia, nausea, vomiting conferring better clinical outcomes as compared to in-center dialysis. It is unclear and somewhat speculative how the lower incidence of complications can be translated into saved money.

## 4. Discussion

There has been increasing interest in encouraging intensification of dialysis regarding frequency and duration [23]. Home hemodialysis has demonstrated superiority regarding clinical outcomes, quality of life, cost-utility/effectiveness and modest superiority regarding survival when compared to conventional thrice-weekly facility hemodialysis (Table 1) [24,25,26,27,28,29,30]. Medicare established a set of incentives to encourage the increased prescription and utilization of home dialysis modality but in our opinion these incentives are inadequate. Consequently, the goal of increase in the number of HHD is far from being achieved.

Many aspects of the HHD program need to be addressed to increase participation. Patient’s awareness of the different treatment modalities before treatment initiation helps to increase the number of HHD candidates. This requires proper training of nephrologists in the area of HHD and establishment of a more robust pre-dialysis educational program. Medicare reimbursement of HHD may be a disincentive to nephrologists who get reimbursed a lower monthly payment when compared to in-center dialysis care and to facilities which will have to manage patients almost daily while receiving payments of a maximum of three sessions weekly. It is unclear how the regulatory body expects the patient to advocate for themselves if they do not know whether the HHD option exists. At the same time, no true financial incentive encourages participation of facilities and medical providers. It is possible that a change in the reimbursement system provided by Medicare may drive an increase in the HHD utilization and prescription. Expanding home modality programs require a substantial amount of planning and could be costlier at the beginning, especially in the case of HHD because of the amount of training required.

For example, it is estimated that water pipe and waste pipe installation costs around $1500. A special electrical circuit is needed that is solely dedicated to the home hemodialysis machine (20 amperes GFI) and its installation will cost an additional $500. The costs of home equipment and supplies are not always covered by the dialysis centers and could be an added out-of-pocket cost to patients. Finally, the electrical and water bill is expected to be higher. These extra costs could be burdensome on patients and may deter them from utilizing this dialysis option. Therefore, the establishment of a program that helps patients with covering the extra costs of therapy or mandating electrical and water companies to offer reduced rates to patients may become an incentive and could potentially increase the utilization of this mode of therapy [31]. However, the payback period is on average estimated to be around 14 months [30]. However, more expenses should be offset easily by increasing productivity, quality of life and lower expenses. Since Medicare is a federal program, it should be relatively easy to change the payment structure to encouraged participation in HHD despite the higher direct cost. In the view of federal regulators, a big picture should be present and that one is in HHD’s favor.

## 5. Conclusions

HHD presents itself as a valid option that lessens the burden already imposed by the ESRD diagnosis and the lifelong commitment of renal replacement therapy. Unfortunately, there has been a documented steady decline in the use of HHD where the point prevalence trend was as high as 6% in 1986 reaching less than 2% in 2010 [32]. While there was a call by key stakeholders to increase the utilization of HHD, barriers remain in place that may continue to preclude the increase of HHD use which are partly attributed to health care and physician factors. The set bundled payment by Medicare is the same for three days per week in-center HD and the more frequent 5–6 days per week HHD. This arrangement limits the approval of HHD to candidates who have employer group health plans as compared to Medicare alone plan, where the latter requires medical justification for the more frequent HD sessions [33]. This surely is burdensome to patients and the providers and their team. Providers with the proper training possess the skills to evaluate and manage prospective HHD candidates. They also tend to have a dedicated team to educate the patients in the pre-dialysis phases which usually contribute to increasing HHD utilization when patients are properly educated toward treatment modality options [7,8,9,10,11,34]. Therefore, not possessing the proper education and training may render the aim of increased HHD utilization a challenging task.

HHD is an option with many clinical and nonclinical benefits but it may also increase the risk of certain complications. There have been mixed results on the survival benefit rendered by HHD compared to In-center HD. However, five out of seven registries reported on survival benefits of HHD as compared to in-center HD [34]. Although somehow inconclusive, HHD has contributed to better quality of life of individuals using this modality compared to in-center modality [19,20,21,22]. While it is an option that increases patient’s autonomy and most importantly HHD presents as employment compatibility, this modality is not for everyone. Patients with a lack of motivation, support groups and proper living conditions added to the presence of major medical and psychological comorbidities are not candidates for the HHD modality. Lastly, the frequent accessing of the vascular site may lead to vascular complications related to technical failure and increase the risk of infection leading to sepsis [35].

In conclusion, this review paper highlighted the many aspects that impact the utilization of HHD. HHD modality is a renal replacement therapy that promises to provide the proper candidates with the sense of empowerment and independence. There exist many barriers that preclude the increase utilization of this option and addressing the different issues at all levels including system, patient and provider levels may contribute to positive changes and increase its utilization as it is hoped by key stakeholders in this field.

## Figures and Tables

**Table 1:** Summary table of Literature Review Findings.

Key Highlights	Summary of Literature Review Findings
**Medicare Payment** a. Bundled Payment System b. Medicare payment for training c. Medicare physician reimbursement	Reimbursement for HD therapy covers up to three sessions weekly [6]. Medicare pays an adjusted rate of $50 per treatment for up to 25 sessions totaling $1881 [5]. Nephrologists receive a one-time payment of $500 for supervising home dialysis training. They receive $237 monthly for HHD patients compared to $266 for in-center patients [5].
**Cost Effectiveness of HHD**	➢ Cost effectiveness ratio of HHD was $4200 as compared to $11,000 for conventional HD. ➢ Annual cost of care difference of $21,000 making HHD cost efficient ($51,252 vs. $29,962) [16,17,18].
**HHD and Clinical Outcomes**	➢ Improved control of hypertension [19,20,22]. ➢ Significant decrease of left ventricular mass at 12 months (16 vs. 2 gm, *p* = 0.001) [19,20]. ➢ Patients had higher hemoglobin levels and required 41% less of erythropoietin agent [21].
**HHD and Quality of Life**	➢ Significant in the physical health composite score (3 vs. 0.2; *p* = 0.004) [19]. ➢ Higher utility score conferring higher quality of life (0.77 ± 0.23 vs. 0.53 ± 0.35, *p* = 0.03) [27].
